# Pilot Studies on Two Complementary Bath Products for Atopic Dermatitis Children: Pine-Tar and Tea

**DOI:** 10.3390/medicines6010008

**Published:** 2019-01-08

**Authors:** Kam Lun Hon, Wing Gi Gigi Ng, Jeng Sum C. Kung, Ping Chung Leung, Ting Fan Leung

**Affiliations:** 1 Department of Paediatrics, The Chinese University of Hong Kong, Hong Kong 00852, China; giging072206@yahoo.com.hk (W.G.G.N.); jsckung@gmail.com (J.S.C.K.); tfleung@cuhk.edu.hk (T.F.L.); 2 Institute of Chinese Medicine, The Chinese University of Hong Kong, Hong Kong 00852, China; pingcleung@cuhk.edu.hk

**Keywords:** atopic dermatitis, bath, pine-tar, green tea extracts, quality of life, sample-size calculation

## Abstract

**Background:** Few standardized bath product clinical trials were performed for atopic dermatitis patients. Pine-tar and green tea extracts are plant-derived products that have been described as having anti-allergic effects which may reduce AD disease severity. **Methods:** The efficacy of two complementary bath products was studied and compared. Efficacy and acceptability of the bath products were measured by patient general acceptability of treatment (GAT: very good, good, fair or poor), disease severity (SCORAD: SCoring Atopic Dermatitis), quality of life (CDLQI: Children Dermatology Life Quality Index), and pertinent clinical parameters were measured before and after four weeks of treatment. Sample size calculations for further clinical trials were performed. In one group, nine AD patients were subjected to bathing with a pine-tar bath oil for 10–15 min daily for four weeks. In another group, 20 AD subjects bathed with a teabag containing green tea extracts for four weeks. **Results:** Significant improvements in clinical- and patient-orientated parameters were found in the pine-tar bathing group, but not the tea-bag bathing group. Both groups reported very good/good GAT on the studied products. Teabag bathing was considered not efficacious for further clinical trials. **Conclusions:** The pilot studies provided preliminary data on the efficacy of pine tar bath oil. We do not document a significant efficacy for bathing with tea extracts. Bathing with pine-tar is potentially a complementary topical treatment with good patient acceptance and adherence, but further evidence-based research for its recommendations is needed.

## 1. Introduction

Atopic dermatitis (AD) is a complex disease involving various degrees of skin inflammation, pruritus, erythema, dryness, and infections [1,2]. The mainstay of treatment for AD is the regular usage of emollient and topical anti-inflammatory medications when needed [1,2,3,4]. Emollients provide an occlusive barrier to retain moisture and to protect the skin from irritants. Emollients have a short- and long-term steroid-sparing effects, besides the established effects of decreasing transepidermal loss of water and skin susceptibility to irritants [5]. Some emollients have been claimed to possess anti-microbial, anti-itch, and anti-inflammatory actions [4,6,7]. They are complex mixtures of chemical agents designed to make the epidermis softer and more pliable [8,9]. Choosing an appropriate emollient has been a major concern for patients and physicians. Despite price differences, the major ingredients of an emollient are similar, consisting of petrolatum, paraffin, glycerin, plant-derived butter, and oils [4,6,7,9]. However, many parents and patients use emollients and topical treatments inconsistently, rendering the management of AD very difficult at times [10]. Complementary and alternative medicine (CAM) has gained popularity in the management of many chronic diseases, including AD [11]. Many CAM products are claimed to be natural and contain herbal ingredients [11]. One modality of CAM is the use of various herbal or plant-derived bath oil for bathing. Studies have reported that regular bathing using green tea extracts markedly reduces disease severity, and hence has been proposed to be an effective and safe treatment for patients with AD [12,13]. Various components of green tea have been reported to have beneficial effects [12,14,15,16,17,18]. The main constituents of green tea are catechins, the biological activities of which include anti-mutagenic, antibacterial, antioxidant, and antitumor properties [12,14,15,16,17,18]. One of the major catechins, Epigallocatechin gallate (EGCG), has been shown as having anti-inflammatory effects in vivo, by suppressing IL6, IL8, and TNF-α expression, and interfering with the IL-1β pathway [19,20]. Animal studies have demonstrated that oral administration of tea (i.e., green, black, or oolong) suppresses type I and type IV allergic reactions [21,22].

In clinical trials, AD patients treated with green tea extracts show a marked clinical improvement of their AD and reduction of pruritus [12,13,23]. Furthermore, the therapeutic efficacy of oolong tea in recalcitrant AD may be the result of the antiallergic properties of tea polyphenols [24,25]. A study reported that 121 patients with recalcitrant AD were instructed to drink oolong tea for six months [25]. After one month of treatment, 63% of the patients showed a marked to moderate improvement of their condition. A good response to treatment was observed in 54% patients at six months. However, subjects had to drink 1 L of Oolong each day. It may be difficult to demand children to drink such an amount of tea each day. Hence, the topical application of tea as in bathing may be an alternative in children with AD. Four patients with AD associated with *M. sympodialis* bathed with green tea extract for four weeks [13]. All patients showed a marked improvement in the mean SCORing Atopic Dermatitis index (SCORAD) and a significant decrease in the mean values of serum eosinophil counts was observed after treatment.

Pine-tar and various tar ingredients have also been used in dermatologic conditions [16,26]. Pine-tar is derived from pine and contains an antiallergic ingredient. Exact therapeutic mechanisms of pine-tar are still unknown, but it is suggested that the high polycyclic aromatic hydrocarbon (PAH) content of tars could be one of the active ingredients that contribute to anti-inflammatory effects and skin barrier repair in AD [26,27]. We previously tested a number of commercial products and noted that patient factors, namely preference and acceptability, might influence the outcomes of topical treatment independent of ingredients in these products [6]. It is noted that parents are very receptive to daily showering and bathing, despite inconsistency with topical treatment. Hence, bathing could offer an alternative therapy for repairing the skin barrier, inflammation, and infections associated with AD. This report describes the methodology in studying patient acceptability and efficacy of bathing for AD, and reports two pilot studies on herbal ingredients for bathing. 

## 2. Materials and Methods

### 2.1. Subject Recruitment

Patients (4–18 years old) suffering from AD attending the Pediatric dermatology outpatient clinic of a university-affiliated teaching hospital were recruited from March 2016 to January 2018. Diagnosis of AD was made according to the criteria proposed by Hanifin and Rajka [28]. AD disease severity was assessed using SCORAD, in which disease activity of AD is divided into three categories. In brief, an objective SCORAD value < 15 points were referred to as “mild”, 15–40 points were referred to as “moderate”, and >40 points were regarded as “severe” [29]. Patients with moderate-to-severe disease were recruited. Both the pine-tar and the teabag bathing studies used the same inclusion criteria. Exclusion criteria included any current or recent use (within the past four weeks) of oral antibiotic preparations and coexisting skin diseases other than AD. Informed written consent was obtained from parents, legal guardians, or patients before recruitment. Ethical approval was obtained from the Joint Chinese University of Hong Kong—New Territories East Cluster Clinical Research Ethics Committee (Ref. CREC: 2015.588 and CREC: 2017.399, 24 August 2017).

### 2.2. Study Design

Both studies were designed as a four-week single-center, one arm, open-label, self-control pilot clinical study. The subjects were evaluated at baseline and four weeks following the study.

In both studies, subjects were asked not to use any medications other than their usual treatment. Patients attending the dermatology clinic were prescribed mometasone furoate 0.1%, twice a week, and chlorphenamine 4 mg or cetirizine 10 mg daily as topical corticosteroid and oral antihistamine, respectively. The amount of medication usage was recorded. Disease severity was measured by SCORAD, Patient Oriented Eczema Measure (POEM), and Nottingham Eczema Severity Score (NESS). Quality of life was measure by Children Dermatology Life Quality Index (CDLQI), and Pediatric Allergic Disease Quality of Life Questionnaire (PADQLQ) [30,31,32,33,34,35,36]. Objective measurements, including skin hydration (SH), transepidermal water loss (TEWL), and erythema, were measured at 2 cm below the right antecubital fossa by Mobile Skin Center MSC 100 equipped with a corneometer CM 825 and Tewameter TM 210 probe (Courage & Khazaka electronic GmbH, Cologne, Germany), MoistureMeterSC, Vapometer, SkinColorCatch (Delfin Technologies Ltd., Kuopio, Finland) [33,37]. Blood markers, including eosinophil %, total IgE, were also measured. *Staphylococcus aureus* status was measured by skin swabbing and bacterial culture at the microbiology laboratory, Prince of Wales Hospital. All these measurements in both studies were performed after the subjects had acclimatized in the consultation room, sitting comfortably in a chair for 10 to 20 minutes.

Patient acceptability of treatment and adverse events such as allergic reactions (acute contact dermatitis, urticaria, and anaphylaxis) and compliance to treatment were recorded for both studies.

### 2.3. Studied Product

For the tea bath study, tea extract was provided by the Institute of Chinese Medicine, The Chinese University of Hong Kong. The extract provided by the Institute of Chinese Medicine was extracted at 65 °C for 50 minutes two times for maximum yields of caffeine and total polyphenol contents (TPC). The extract was dried with a spray drier into powder [38,39]. The concentration of tea extracts in the bath solution was 0.02%. Four grams of tea extract powder was sprinkled into a large plastic tub (total volume 38 L), and then 20 L of warm water was added. Subjects were instructed to bathe with dilute tea extract for 15 minutes each night for four weeks. Bath temperature was recommended to be maintained at 37–40 °C. For the pine-tar bath study, subjects were assigned to use a proprietary pine-tar bath oil 2.3% *w*/*w* (Pinetarsol, Ego Pharmaceuticals; Australia). Subjects were instructed to bathe with 15–20 mL of the 2.3% pine-tar bath oil for 15 minutes daily in tepid water, according to the manufacturer’s instruction. 

### 2.4. Statistical Analysis

Numerical data were expressed as the median and interquartile range (IQR). SCORAD and Quality of Life score change from baseline in the symptom score were assessed within treatment groups by using a paired *t*-test or Wilcoxon test to compare the changes between pre- and post-treatment. Proportional data were analyzed using the Chi-square test. All comparisons were two-tailed, and *p* values equal to or less than 0.05 were considered statistically significant. The statistical analyses were made with SPSS for Windows 25.0 (IMB Corp., Armonk, NY, USA). 

## 3. Results

Twenty subjects (40% male, mean age: 10.8; IQR) and nine subjects (55.6% male, Median age: 10.3; IQR: 7.1–13.9 years) with moderate to severe AD took part in the tea bath study and pine-tar bath study, respectively, for four weeks (Table 1). The teabag bathing study was conducted in Mar 2016 to May 2017, and the pine-tar bathing study was conducted between Oct 2017 and Jan 2018. 

The subjects recruited in both studies suffered from moderate to severe eczema. They were assessed by the same severity scores, including POEM, NESS, and quality of life score (CDLQI). Blood markers, including complete white blood cell count and serum IgE, were measured in both studies. Objective dermatology parameters including skin hydration (SH) and TEWL were measured using non-invasive devices from different manufacturers. Both sets of skin devices provided reliable and repeatable readings. The skin measurements were performed by different set of devices and therefore should not be used for direct comparison, yet both demonstrated comparable readings [33]. Both studies used a non-parametric equivalent of the paired-t-test for statistical analysis, allowing evaluation of the results independently.

Disease severity (objective SCORAD) did not display any statistically significant changes after a four-week tea-bag bathing treatment. On the other hand, disease severity (objective SCORAD) improved after the pine-tar bath treatment (Table 2; *p* = 0.050). There were also significant improvements in POEM (*p* = 0.021), CDLQI (*p* = 0.011), and PADQLQ scores (*p* = 0.036) in the pine-tar bath study. Skin measurements, including SH, TEWL, or erythema; blood markers (eosinophil % and IgE); and *S. aureus* status did not exhibit statistical improvements in either study (Table 2).

Compliance in both studies was good. Subjects generally managed to use the bathing materials daily as instructed. For the tea bath study, 70.0% reported “very good” or “good”, whereas 30.0% reported “fair” or “poor”, acceptability; while for the pine-tar bath study, 77.8% reported “very good” or “good”, whereas 22.2% reported “fair” or “poor”, acceptability (Table 2). 

No serious adverse effects were reported during the study, with individual reports of a ‘tingly’ sensation by some subjects when applied to inflamed skin.

**Figure 1:**
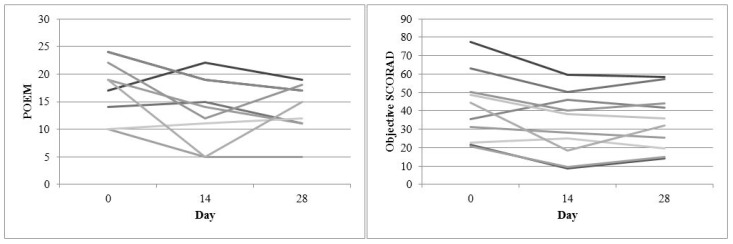
Disease severity change over four weeks (POEM and Objective SCORAD) for the pine-tar bath study.

Change in disease severity over four weeks (POEM and Objective SCORAD) for the pine-tar study was shown in Figure 1. Each line represents one subject. POEM demonstrated a fluctuating disease severity over four weeks, with a non-significant correlation (Day 0 and 14: B = 0.69, *p* = 0.062; Day 0 and 28: B = 0.14, *p* = 0.703). There was a more consistent improvement in the objective SCORAD over week 2 and week 4 (Day 0 and 14: B = 0.86, *p* = 0.001; Day 0 and 28: B = 0.95, *p* < 0.001). 

Based on the findings of this pilot study on pine-tar, we obtained an effect size of 8.4 (reduction in objective SCORAD score). Based on our previous studies, the SD for objective SCORAD was around 12 [40,41]. Considering both effect size and SD, we obtained a standardized effect size (effect size/SD) of 0.725. By referring to Table 3, taking the probability of type I error: α (two sided) to be 0.05 and probability of type II error: β to be 0.20 (1–0.80), around 34 participants per group would be required for a future randomized controlled trial (Table 4). By using a cross-over approach, each subject would be his/her own control. Anticipating a potential drop-out rate of around 10–15%, a sample size of around 40 subjects would be sufficient to demonstrate the efficacy of our bathing treatment. 

## 4. Discussion

We demonstrated that the pine-tar product is efficacious in reducing eczema severity following a four-week usage, despite a small recruitment size. There was also an improvement in patient-oriented measures of POEM, CDLQI, and PADQLQ. Conversely, no statistical significance was demonstrated in any of the study parameters in the teabag trial despite a larger recruitment size. Tar was an ancient Greek treatment for skin diseases, such as atopic dermatitis and psoriasis [42]. Similar to coal tar, pine-tar consist of complex hydrocarbons mixtures, with polyaromatic hydrocarbons (PAH) being one of the major groups [42]. In vivo studies with coal tar have shown that PAHs would activate the aryl hydrocarbon receptor (AhR) pathway with beneficial downstream effects in skin keratinocytes [43]. Similar experiments have been repeated with soybean tar (glyteer) by a Japanese group and found that PAH from glyteer rescued the T-helper-mediated downregulation of filaggrin expression via AhR and improved AD conditions [44]. Although in vivo and in vitro studies of pine-tar are lacking, our pilot study demonstrated the efficacy of pine-tar in AD management. Hence, we hypothesise that the active ingredient (PAH) in pine-tar functions in a similar fashion. Bench and clinical studies are underway to investigate the hypothesis.

Tea has been postulated to have anti-inflammatory effects. A Japanese case series demonstrated that the consumption of one litre of Oolong tea helped ameliorate AD [25]. However, it is not practical to advise a child with AD to drink one litre of tea each day. We postulate that a topical bath with a tea extracts in a teabag may have similar efficacy, without subjecting the child to drinking a large amount of tea each day. However, very good/good acceptability, but not efficacy was demonstrated. One possibility is that the strength or concentration of tea ingredient was too weak at the level of the target organ (i.e., skin), but the bathing tea concentration was already higher than that of the oral consumption of tea. A few research directions may be considered based on this pilot study on tea bath. In future testing, the strength of tea extracts in the bath could be increased. Various types of tea, such as Oolong, green tea, and black tea, should also be investigated for the difference in efficacy for treatment. Tea might need to be consumed orally rather than topically to replicate potential efficacious effects claimed in previous trials for children with AD.

Study design is important in the evaluation of efficacy of proprietary products. From the pine-tar study results (Figure 1), the objective SCORAD is suggested to be a reliable severity score for evaluation. A recent study had reported a negative finding for emollient bath additives for AD treatment [45]; however, the study only used POEM as a severity score, and the subjects were also suffering from a milder disease. In the present pine-tar study, subjects who suffered from chronic AD with moderate severity seemed to benefit from the pine-tar bath. The pine-tar demonstrated comparable improvement from week 2 to week 4. A randomized controlled trial is currently underway to confirm the efficacy of pine-tar bathing observed in this pilot study. 

Reports on proprietary emollients commonly described their efficacy and adverse effects profiles in AD treatment [3,8,46,47]. Very few studies have reported on patient’s acceptability or satisfaction of the trial emollient [6,48]. Likewise, there have been very scanty reports on efficacy and patient acceptability of bathing for AD [4]. Most published reports are on the effects of adding bleach instead of bath oil in bathing for the treatment of AD [4,40,49]. Patient’s acceptability is important in a physician’s recommendation. Acceptability may be translated into adherence and efficacy [6]. Our group has previously conducted a study evaluating the moisturizing practice and preference of patients with AD and found that the doctor’s recommendation was the major source of advice when choosing an emollient or proprietary product. Physicians should therefore be knowledgeable about proprietary products for AD patients and their relevant research [4].

## 5. Conclusions

The efficacy profile of a pine-tar bath oil has been described. Additional evidence of the efficacy and potential mechanisms of the pine-tar bath will need to be further studied. Clinical efficacy of pine-tar bathing should be confirmed by randomized trials with its vehicle. We do not document any efficacy for bathing with tea ingredients. Bathing with pine-tar is potentially a complementary topical treatment with good patient acceptance and adherence, but further evidence-based research for its recommendations is needed.

## Figures and Tables

**Table 1:** Patient demographics of the Tea bath (*n* = 20) and Pine-tar bath (*n* = 9) study.

Patient Information	Median (IQR) for Tea Bath Study	Median (IQR) for Pine-Tar Bath Study
**Sex (%)**	8 (40.0) Male 12 (60.0) Female	5 (55.6) Male 4 (44.4) Female
**Age (Years)**	10.8 (7.5–15.7)	10.3 (7.1–13.9)
**Objective SCORAD (SCORAD without the subjective components)**	33.3 (24.8–45.5)	40.8 (22.2–56.6)
**POEM**	18.5 (13.0–23.0)	17.7 (12.0–23.0)
**NESS**	15 Severe; 2 Moderate	7 Severe; 2 Moderate
**CDLQI**	9.0 (6.0–16.0)	11.0 (10.0–16.5)
**PADQLQ**	-	50.0 (24.5–54.0)
**SH (a.u.)**	18.7 (14.7–31.5)	10.2 (7.6–14.7)
**TEWL (g/m^2^/h)**	7.1 (5.3–11.9)	13.5 (7.4–18.0)
**Erythema(a.u.)**	-	414.0 (393.0–418.5)
**Eosinophil %**	7.0 (4.0–10.0)	8.0 (6.3–9.8)
**IgE (IU/mL)**	3581.5 (1650.5–5894.5)	3169.0 (1041.5–8181.5)
**Log [IgE]**	3.5 (3.2–3.7)	3.5 (3.0–3.9)
**S. aureus colonization (Number, %)**	12 (60.0)	3 (42.9)
**Days using topical steroid/week**	3.0 (1.0–7.0)	2.0 (0.0–2.0)
**Days using oral anti-histamines/week**	2.0 (0.0–6.5)	-

a.u.: arbitrary unit.

**Table 2:** Demographic changes pre- and post-study in the Tea bath and Pine-tar bath study.

	Tea Bath Study			Pine-Tar Bath Study		
	Pre	Post	*p*-Value	Pre	Post	*p*-Value
**Objective SCORAD**	33.3 (24.8–45.5)	29.9 (16.4–37.4)	0.324	40.8 (22.2–56.6)	32.1 (17.4–50.7)	**0.050**
**POEM**	18.5 (13.0–23.0)	16.5 (12.0–23.0)	0.393	17.7 (12.0–23.0)	15.0 (11.0–17.5)	**0.021**
**CDLQI**	9.0 (6.0–16.0)	10.5 (8.0–19.0)	0.305	11.0 (10.0–16.5)	8.0 (4.0–11.5)	**0.011**
**PADQLQ**	-	-	-	50.0 (24.5–54.0)	29.0 (19.0–41.0)	**0.036**
**SH (a.u.)**	18.7 (14.7–31.5)	18.5 (14.1–34.6)	0.575	10.2 (7.6–14.7)	18.0 (11.4–20.5)	0.192
**TEWL (g/m^2^/h)**	7.1 (5.3–11.9)	8.8 (5.2–13.3)	0.970	13.5 (7.4–18.0)	10.7 (9.4–21.1)	0.441
**Erythema (a.u.)**	-	-	-	414.0 (393.0–418.5)	401.0 (396.5–415.5)	0.722
**Eosinophil %**	7.0 (4.0–10.0)	7.0 (4.0–9.0)	0.858	8.0 (6.3–9.8)	7.0 (7.0–10.5)	0.172
**IgE (IU/mL)**	3581.5 (1650.5–5894.5)	3277.0 (1611.0–6175.0)	0.814	3169.0 (1041.5–8181.5)	2355.0 (948.5–9032.8)	0.141
**Log [IgE]**	3.5 (3.2–3.7)	3.5 (3.2–3.7)		3.5 (3.0–3.9)	3.3 (3.0–4.0)	
**S. aureus status (no growth)**	8 (40.0)	9 (45.0)	0.257	3 (42.9)	6 (66.7)	0.386
**GAT**						
**Very good or Good**		14 (70.0)		7 (77.8)		
**Fair or Poor**		6 (30.0)			2 (22.2)	

Disease severity and patient self-reported questionnaire scores improved significantly after the pine-tar bath treatment (*p*-value ≤ 0.05). Skin measurements, blood markers, and *S. aureus* status did not exhibit any statistical improvements in either study. General acceptability of treatment (GAT) for Tea bath and Pine-tar bath study was good. Median and IQR for numeric values. a.u.: arbitrary unit.

**Table 3:** Sample size required per group.

One-Sided α=	0.005			0.025			0.05		
Two-Sided α=	0.01			0.05			0.10		
E/S* β=	0.05	0.10	0.20	0.05	0.10	0.20	0.05	0.10	0.20
0.10	3565	2978	2338	2600	2103	1571	2166	1714	1238
0.15	1586	1325	1040	1157	935	699	963	762	551
0.20	893	746	586	651	527	394	542	429	310
0.25	572	478	376	417	338	253	347	275	199
0.30	398	333	262	290	235	176	242	191	139
0.40	225	188	148	164	133	100	136	108	78
0.50	145	121	96	105	86	64	88	70	51
0.60	101	85	67	74	60	45	61	49	36
0.70	75	63	50	55	44	34	45	36	26
0.80	58	49	39	42	34	26	35	28	21
0.90	46	39	32	34	27	21	28	22	16
1.00	38	32	26	27	23	17	23	18	14

**Table 4:** Summary of steps for sample size calculation.

Steps	Calculation Example using Pine-Tar Study
Step 1—Determine effect size from pilot study:	Effect size = 40.8 − 32.1= 8.4 (obj. SCORAD score)
Step 2—Determine SD of outcome variable	SD = around 12 **
Step 3—Determine Standardized effect size	Standardized effect size = effect size/SD = 8.4/12 = 0.725
Step 4—Decide α (probability of Type I error) and β (probability of Type II error)	α (two sided) = 0.05; β = 1 − 0.80 = 0.20;
Step 5—Find out expected sample size per group from Table 3	Using Table 3, around 34 participants per group would be required for a future randomized controlled trial.

**: value comes from our research databank (raw data not shown in this paper).
